# The Origin of Cow Pox and the Nature of the Vaccine Virus

**Published:** 1864-09

**Authors:** 


### THE ORIGIN OF COW POX AND THE NATURE OF THE VACCINE VIRUS.

Investigation on this subject in the Paris Academy of medicine, has led to the following conclusions:—

1st. That vaccine virus (as a thing separate and apart) has no existence.

2d. That the pretended vaccine virus, which we consider antagonistic to, and neutralizing variola, is the variolus virus itself.

3d. That the equine and bovine species are subject to an eruptive malady which is identical, as regards its nature, with variola of the human species.

4th. It is demonstrated that the same is the fact as regards several other species of animals, pigs, sheep, dogs, goats. apes, etc.

5th. The local and general phenomena with animals are the same as those observed in man. The only difference as regards the pustules are those which depend on the structure of the skin and the number of the hairs.

6th. As in the human species, so in the equine and bovine, variola may appear sporadically or epidemically.

7th. From the horse we may inoculate the cow, and reciprocally.

8th. From the cow we may inoculate, without difficulty, individuals of the human species, provided they have not had spontaneous or inoculated variola.

9th. The cow, the horse, and several other species may be inoculated with variolus matter from the human species.

10th. When a variolus epidemic occurs among men, it often extends itself, by contagion, to other animals.

11th. An epidemic of variola may commence among animals and extend to man.

12th. Inoculated variola produces a much less degree of general reaction than does variola developed by contagion. This is true in both man and lower animals.

13th. The pustules which result from inoculated vairola, are often limited to the points inoculated.

14th. When a secondary eruption is produced, it is almost always insignificant, and composed of a small number of pustules.

15th. In a general manner we may say that the vairola of animals is more discreet, and less severe, than that of the human species.

16th. That the dangers of inoculation of variola in man have been much exaggerated. The unprejudiced study of what has been written on this subject will convince of this.

17th. It is probable that animals, as man, are subject to apthous eruptions

18th. But the *maladie aptheuse*, as it is described by writers on veterinary medicine, is nothing less than variola.—*Medico Chirurgical Review.*—*Pacific Medical and Surgical Journal.*

